# Positioning the Sense of Coherence (SOC) in Disaster Recovery Planning and Design

**DOI:** 10.3390/ijerph22020161

**Published:** 2025-01-25

**Authors:** Cornelius Ayodele Ojo, Traci Rose Rider

**Affiliations:** College of Design, North Carolina State University, Raleigh, NC 27695, USA; traci_rider@ncsu.edu

**Keywords:** sense of coherence, conservation of resources, disaster resilience, climate resilience, salutogenesis, adaptive resilience, stress coping, social equity, urban health and well-being

## Abstract

“Whence the strength?” This compelling question, posed by Aaron Antonovsky in 1979, sets the stage for understanding the role of sense of coherence (SOC), a human-focused psychosocial concept, in fostering resilience amidst escalating climate-induced disasters such as hurricanes, floods, and earthquakes. This paper is the first step in a larger research agenda aimed at exploring how the human experience of disasters, guided by Antonovsky’s SOC framework, can be better integrated into disaster recovery planning and design, laying the theoretical foundation for subsequent studies. This paper examines which supports help people stay resilient during disasters, focusing on the role of SOC in recovery. By integrating Antonovsky’s SOC concept with Hobfoll’s Conservation of Resources (COR) theory, it also draws from other published works on stress and disaster recovery to explore how disaster recovery planning and design can be improved. The findings indicate that the post-disaster recovery phase presents a critical window for implementing policies that address vulnerabilities in disaster-prone communities and enhance long-term resilience. Methodologically, this paper advocates for an interdisciplinary approach, suggesting that both quantitative and qualitative insights are vital for capturing human experiences in disaster contexts. Ultimately, this paper presents a framework for integrating human dimensions of resilience into disaster recovery planning.

## 1. Introduction

Maslow’s Hierarchy of Needs provides a vivid description of human needs from which disaster managers and policymakers can learn. This framework illustrates that human needs exist at different levels—from fundamental survival requirements to higher-order aspirations for self-fulfillment [1]. This structure underscores that, while basic survival—especially during disasters—forms the foundation of human needs, individuals require more than physiological safety to achieve wholeness and well-being. Traditionally, disaster preparedness and recovery efforts have concentrated on hazard mitigation and vulnerability reduction. However, limiting planning for these aspects restricts survivors’ potential to attain higher psychological well-being, social belonging, esteem, and self-actualization during and between disasters.

Effective disaster recovery planning should facilitate a progression up Maslow’s pyramid, fostering environments where health transcends mere survival. This comprehensive approach enables individuals to regain control, find meaning, and build social connections, establishing the foundation for long-term resilience. Such an understanding aligns with Antonovsky’s salutogenic model of health, which emphasizes the importance of developing an individual’s sense of coherence (SOC)—a measure of their capacity to navigate and manage stressful situations [2,3].

Antonovsky [2,3] provide a complementary lens to view human health and well-being, especially in communities prone to climate-induced hazards, moving from merely combating these stressors to adapting and thriving despite their prevalence [2,3]. This novel perspective has significant implications for disaster recovery planning, particularly through the concept of SOC, which reframes recovery as an opportunity for growth rather than merely a return to a baseline. Antonovsky identified three fundamental principles that enhance this understanding: first, the principle of the Ubiquity of Pathogens and Stressors, which posits that stressors are inherent in the human environment, necessitating effective coping mechanisms to maintain health [2,4,5]; second, the Limitations of Pathogenic Research, which argues that a sole focus on disease origins and risk factors limits our understanding of health and resilience [6,7,8,9]; and third, the SOC concept, which is critical for understanding how individuals adaptively comprehend, manage, and derive meaning from stressful situations [2,3,4,10].

These insights from Antonovsky’s work compel a reimagining of disaster management and recovery through design and planning perspectives. Despite the increasing acknowledgment of SOC’s importance in health and resilience literature, comprehensive studies integrating SOC into disaster recovery planning and design remain scarce. The current research either predominantly focuses on the quantitative use of the SOC in disaster contexts [11,12] or addresses immediate disaster response and physical reconstruction, often neglecting the psychosocial dimensions vital for long-term adaptive resilience [13,14,15]. This paper addresses that gap by exploring how the concept of SOC can inform design-based strategies for enhancing disaster recovery amidst escalating climate-induced threats.

Three objectives are addressed in this paper. First, it establishes natural hazards as acute stressors that impose both immediate and long-term demands on affected communities [2,8]; second, it clarifies the distinction between natural hazards and disasters; and third, it frames disasters as focusing events that create post-disaster opportunities for building resilience [16,17,18,19,20]. As such, this paper advocates for a paradigmatic shift in disaster planning and design—one that prioritizes strengthening the sense of coherence in individuals and communities. This approach offers a path toward enhanced resilience and adaptivity in the face of both emerging and persistent environmental risks.

## 2. Methodology

This study employs a literature synthesis approach, focusing on key theoretical frameworks and recent research related to health, stress, and adaptive resilience. The analysis draws exclusively on published works, including foundational texts and peer-reviewed articles. No primary or secondary data were collected for this study; rather, the findings are derived from a structured analysis of existing literature. The analysis is grounded in four foundational texts:

i. *Health, Stress, and Coping: How People Manage Stress and Stay Well* by Aaron Antonovsky (1979) [2].
ii. *Unraveling the Mystery of Health* by Aaron Antonovsky (1987) [3].
iii. *Ecology of Stress* by Stevan E. Hobfoll (1988) [21].
iv. *Lessons of Disaster: Policy Change After Catastrophic Events* by Thomas A. Birkland (2006) [17].

These works were chosen for their seminal contributions to understanding health, stress, resilience, and disaster-related policy changes.

Other sources of data included citation mining, reference mining, and a database search. First, recent publications citing these foundational works were identified via citation mining on Google Scholar and ResearchRabbit to select those that explicitly connect stress, health, and adaptive resilience. Then, the further mining of the references cited in the identified publications was conducted to uncover additional relevant studies. Finally, to ensure a comprehensive review, additional searches were conducted on ProQuest Central and Academic Search Complete (via EBSCO) to identify relevant papers published between 2014 and 2024. Boolean search phrases included “stress”, “health”, “resilience”, “natural disaster”, “resistant resources”, “Policy”, “design”, and “planning”. Abstracts and full texts were then screened for relevance to the interplay of stress, health, and resilience before inclusion in the study. Initially, abstracts were reviewed to eliminate studies that did not align with the objectives of this paper. For those abstracts that were relevant, the full texts were then assessed to determine their contribution to understanding the key themes of stress, health, and resilience. Studies that did not offer direct insights into these intersections were excluded from the final synthesis.

## 3. Results

A total of 1469 results were obtained from the citation and reference mining and database search. All were imported into Zotero, and after the title screening, the number was reduced to 219. This was swiftly followed by abstract screening, which further reduced the number to 169. Ultimately, a total of 63 publications (journal articles and books) informed the content of this paper.

### 3.1. Disentangling the Nature of Stressors

Life is full of stress, according to Selye [22]. He suggested that stress is a constant part of living. Antonovsky [2,3] and Gunderson and Holling [23] agree with this idea by challenging the traditional belief that humans encounter stressors sporadically, suggesting instead that every individual is continuously immersed in a metaphorical “polluted river” of life, shaped by historical, socio-cultural, and environmental factors. Some stressors are natural, others are man-made whether directly or indirectly. Consequently, the question becomes how individuals and communities can effectively manage the constant and, at times, intense, onslaught of stressors while maintaining their health and well-being.

Stressors are any tension-inducing objects, events, situations, or occurrences capable of making demands, whose responses may or may not be readily available [2,3], or conditions of threats, challenges, demands, or structural constraints that call into question the operating integrity of an organism [24]. Stressors can originate from internal or external environments, often distorting the balance needed to maintain normalcy and well-being. The temporal or permanent nature of these imbalances from stressors depends on the ability of the system to respond to the demands of the stressors [2,4,24].

The presence of stressors is an inherent and indispensable aspect of human existence. According to Antonovsky [2], a certain level of disturbance or tension is not just inevitable, but also a fundamental requirement as individuals engage actively with their environments. Antonovsky called this phenomenon “heterostatic (dynamic) equilibrium”, which describes humans as open systems that continuously interact with internal and external factors. This interaction helps people strive for “negentropy” (negative entropy), an ideal state where the demands of internal and external stressors are balanced with the ability to respond effectively [2,3]. In essence, heterostatic equilibrium is a process of consistent and dynamic negentropy.

Inherently, stressors in manageable quantities are not bad. In fact, they serve as positive catalysts for the effective functioning of human systems. However, when stressors become overwhelming or exceed an individual’s capacity to cope effectively, the body enters a state of extended or chronic stress, potentially leading to adverse health outcomes [2].

### 3.2. Natural Hazards as a Form of Stressor

Stressors in human and natural systems can be continuous or periodic, manifesting as routine stimuli, chronic conditions, or acute events [2,4,24]. Natural hazards, such as hurricanes, earthquakes, and floods, represent acute stressors that periodically disrupt equilibrium. These disruptions create tension, causing immediate damage and triggering a cascade of additional stressors, known as “stress proliferation” [25]. Stress proliferation can compromise the ability of individuals and communities to manage everyday life [24,26], significantly reducing well-being by destabilizing the heterostatic equilibrium.

All stressors, whether natural or man-made, routine, chronic, or acute, although fundamentally different, share certain commonalities: they can escalate into full-blown disasters when there is a disconnect between the demands they impose and the community’s capacity to respond. This means that stressors, regardless of their origin, can escalate into full-blown disasters under certain underlying conditions. Furthermore, natural hazards differ fundamentally from disasters. Disasters occur when a natural hazard places demands on a community that lacks the capacity to respond. The extent of this incapacity determines the scope of both immediate and long-term consequences [13]. In this sense, natural hazards become disasters due to their impact on human systems, either directly or indirectly, exacerbated by existing vulnerabilities and preparedness gaps [27].

As purported by Kasperson, the risk associated with any stressor has both technical and social dimensions [28]. Well-thought-out planning, therefore, must help communities recover effectively in all aspects, including technical and social dimensions. Poorly planned recovery efforts risk creating secondary disasters and compounding the stressors faced by affected communities. In contrast, well-planned recovery fosters stability and resilience, helping communities handle enduring challenges [27]. Hence, while natural hazards are inevitable, their transformation into disasters—and the subsequent effectiveness of recovery—hinges on the preparedness and adaptive capacity of the community [24,29].

### 3.3. The Health-Resilience Conundrum

**Figure 1:**
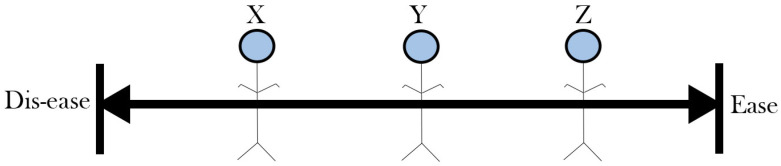
Health as a continuum (adapted from Antonovsky’s salutogenic model of health [2]).

Health and resilience are key concepts to understanding stress responses and well-being in systems. The most widely adopted definition of health is from the World Health Organization (WHO), which defines health as a state of complete physical, mental, and social well-being and not merely the absence of disease or infirmity [30]. This perspective emphasizes a dichotomy, that is, being perfectly healthy or ill. Recent studies, however, show that health is more like a continuum where one can be at different levels of well-being [2,3,31,32,33]. From Figure 1, persons named X, Y, and Z can assume different positions on the health continuum, between ease and disease, depending on their respective ability to manage tensions imposed by stressors. To interpret health and well-being from a dichotomous perspective would mean to overlook this spectrum of possibilities.

From Figure 1, person Z is healthier than Y and Y is healthier than X, but it does not necessarily mean that Z is entirely free of challenges or that X is completely unwell. Everyone on the continuum is faced with tensions from stressors in different forms, but a key factor that determines their positions is the capacity to respond to these imposed tensions. This includes their access to resources, coping mechanisms, and overall ability to maintain balance amidst challenges. Viewing health as “either healthy or sick” oversimplifies the complex ways people experience and manage health.

Central to the shift from a dichotomy to a continuum is the notion of stress. Stress, as conceptualized by Hobfoll [21], is linked to survival and adaptation. The stress mechanism involves a series of dynamic processes through which individuals respond to challenges and adapt to changing circumstances to maintain well-being. Inherently, what facilitates this capacity to respond to and recover from challenges is resilience. Resilience refers to how individuals and systems recover from stressors, whether acute or chronic.

Different types of resilience exist. Engineering resilience focuses on returning to a prior state after a disturbance, while ecological resilience emphasizes the capacity to absorb change without losing essential functions [23,34]. However, adaptive resilience stands out as particularly significant. Unlike the other forms, adaptive resilience extends beyond resistance or recovery by promoting active learning, flexibility, and growth. This type of resilience enables individuals and systems to not only endure challenges but also to evolve and improve in response to them, thereby, fostering sustained well-being [35,36]. Understanding resilience through this adaptive lens is essential for addressing complex challenges such as natural disasters and societal disruptions, where the ability to bounce back and adapt well is imperative to maintaining health and well-being.

As health research and practice continue to evolve, traditional views on health and illness are expanding to include innovative paradigms that provide new insights into well-being and resilience. Two contrasting frameworks stand out in this shift. The pathogenic paradigm, which has long dominated health research, focuses on identifying the causes of diseases and their treatment. In contrast, the salutogenic paradigm offers a complementary approach by exploring the factors that promote health and well-being, rather than solely addressing illness [2,10].

This paper will emphasize the salutogenic approach, as it aligns with the goal of exploring how adaptive resilience supports long-term health and recovery rather than just surviving natural disasters.

### 3.4. The Salutogenic Model of Health

According to Antonovsky [2], to continue to investigate why people fall sick, while noble and important, would mean investigating a phenomenon with endless and continuous possibilities. Shifting the focus, however, even if partially, to studying why some people thrive despite the pervasiveness of pathogens could be the key to unleashing the potential for more people to live well despite the chaotic nature of the world. Antonovsky captured this perspective by stating the following:

“If, then, we can begin to understand this mystery—the mystery of survival, the mystery of why some people’s health is such that they go through life for some of the time with relatively little pain and suffering—we might begin to think about applying this understanding to reduce pain and suffering among the rest of us.”

[2]

Essentially, Antonovsky advocates for a salutogenic perspective that transcends the impact of individual stressors. This broader approach offers a fundamentally different paradigm from the traditional pathogenic model. The key differences between these two approaches are summarized in Table 1 below:

Salutogenesis poses the question: What makes people healthy? Antonovsky believes that the answer lies in a sense of coherence (SOC)—a psychosocial construct that reflects people’s pervasive, enduring, and dynamic confidence in their ability to understand what is happening around them, manage these situations, and find meaning in these challenges [2,3]. Antonovsky posits that individuals with a strong sense of coherence are better equipped to respond to stressors, thereby promoting their overall well-being.

Nascent health research agrees with Antonovsky. Modern perspectives on health and resilience are increasingly recognizing the interplay between personal and social characteristics in managing stress and maintaining health amidst persistent challenges [37,38,39]. Notable are the works of Kaplan [40] and Hobfoll [21], who believe that resilience hinges on an individual’s ability to interpret stressful demands toward achieving valued states or goals. Thus, the human perception of risk and the capacity to adapt, shaped by values, emotions, and personal aspirations, are both valid and legitimate. This viewpoint is further supported by earlier works by Slovic, Kasperson, and Beck, who validate the influence of these subjective factors on resilience and risk management [28,41,42,43]. In addition, Erikson and Peek [13] draw attention to the “utter sense of disorientation” that people experience in the aftermath of disasters, which often compromises recovery. Collectively, these works highlight the substantial psychosocial challenges inherent in disaster recovery and resilience-building.

The concept of the sense of coherence (SOC) thus emerges as particularly relevant in disaster recovery efforts, offering a way to account for, understand, and cater to the enduring psychological, emotional, and social impacts borne by people affected by natural disasters [3,13].

### 3.5. The Sense of Coherence (SOC)

#### 3.5.1. Meaning of the Sense of Coherence (SOC)

According to Antonovsky [3], the key to health despite chronic or critical stressors lies in the perception of one’s environment as coherent, called a sense of coherence (SOC). Antonovsky defines it as follows:

“a global orientation that expresses the extent to which one has a pervasive, enduring though dynamic feeling of confidence that (1) the stimuli deriving from one’s internal and external environments in the course of living are structured, predictable, and explicable, (2) the resources are available to one to meet the demands posed by these stimuli; and (3) these demands are challenges, worthy of investment and engagement.”

[3]

Antonovsky’s concept of sense of coherence (SOC) suggests that individuals with a strong sense of coherence have a broad confidence in confronting life’s challenges, emphasizing the importance of perceived stability and coherence within their circumstances. This means that, while problems are unavoidable, thriving individuals and communities depend not solely on the nature of the problems but importantly on the perceived stability and consistency within those circumstances. Thus, the SOC embodies the belief that, despite challenges and stressors, things are in control, either through personal agency or trusted sources, contributing to a structured, predictable, and explainable environment—even in times of critical distress. This concept, however, differs from an absolute feeling of control, as Antonovsky explains as follows:

“The crucial issue is not whether the power to determine such outcomes lies in our own hands or elsewhere. What is important is that the location of power is where it is legitimately supposed to be. This may be within oneself … the family, patriarchs, leaders, formal authorities …, or a deity”

[2]

In essence, a high sense of coherence (SOC) does not imply an absence of problems or illness. Instead, an SOC is persistent confidence that, in the long term, assures individuals and communities that resources are available to address these problems—whether through individual efforts or the support of others. This enduring feeling of confidence plays a pivotal role in determining the position of the individual or community on the health continuum—either maintaining the current state or nudging us closer to either end.

#### 3.5.2. Components of the Sense of Coherence (SOC)

Antonovsky’s sense of coherence (SOC) consists of three key elements: comprehensibility, manageability, and meaningfulness [2,3]. Comprehensibility, the cognitive part of the SOC, refers to the degree to which the demands from internal and external stressors are structured, predictable, and explicable. Comprehensibility would be similar to knowing the rules of a game, so there are no surprises [2]. For instance, when a coastal community understands the science behind hurricanes, recognizes warning signs, and is familiar with the typical progression of these storms and their potential impacts, the comprehensibility can be said to be high. Essentially, this concept is about understanding the nature of the stressors facing an individual, community, or system, as well as the demands they impose.

Manageability, on the other hand, is about evaluation. The concept of manageability pertains to the resources available to an individual to meet the demands posed by stressors [2]. After understanding the demands placed by internal and external stressors, an individual must be able to identify resources either within their control or accessible through legitimate means to engage these stressors’ demands. For example, when the coastal community mentioned above has established emergency protocols, stockpiled necessary supplies, and trained local response teams to handle various hurricane scenarios effectively, the community is reinforcing its manageability. In essence, when people believe that they have what they need to face challenges, they feel more in control and less stressed.

Lastly, meaningfulness is about motivation. The concept of motivation involves the extent to which one finds a sense of meaning in the stressors’ demands that one is experiencing [2]. Motivation reflects the degree to which an individual, community, or system perceives that these demands are challenges worthy of investment and engagement. The motivational element of the SOC is critical as it drives the willingness of individuals or communities to confront and manage stressors [2,3]. When residents of the coastal community view hurricane preparedness as a critical part of their coastal identity, a means to strengthen communal bond, a source of pride, and a defining characteristic of their community, they are likely to be motivated to engage in activities that foster resilience.

Meaningfulness is deeply tied to personal and collective identity, making it difficult to change, as it is embedded in belief systems. Identities often provide a framework for understanding the world and guide how individuals perceive themselves and their relationships with others [44]. Understanding the intrinsic and extrinsic motivations of individuals and communities is important for the other components of the SOC.

From this background, it is clear that it is not the absence of stressors that makes people healthy, but rather the extent of their coherence amidst these stressors. In other words, the stresses people feel when faced with problems are actually related to a perceived lack of understanding of the nature of the problem stressors’ demands, a perceived lack of resources to engage with those demands, and a perceived lack of understanding of why the demands are even worthy of engagement in the first place.

The SOC, according to Antonovsky, is a unified concept, meaning that the three components—comprehensibility, manageability, and meaningfulness—are all connected [3]. If one of these components weakens, it impacts the others. For example, if a person does not understand a stressor (low comprehensibility), it might make them feel less capable of handling it (low manageability) and less motivated to deal with it (low meaningfulness). In the light of this, all components must be considered holistically [3].

The SOC has been found to significantly predict adaptation and well-being in different cross-cultural settings [45,46]. To effectively strengthen the SOC, exploring the role of the Resistance Resources (RRs) becomes essential. These resources, categorized as both specific and generalized, play a fundamental role in the maintenance and fortification of an individual’s SOC [3]. To explain the nature of Resistant Resources, Hobfoll’s Conservation of Resources (COR) theory [21] is brought into this analysis for additional framing. The COR theory suggests that people try to conserve and protect resources that help them cope with stress. When people have access to strong resources, they can better maintain their SOC and, as a result, adapt to challenges and improve their well-being.

### 3.6. Resource Mobilization and SOC Strengthening: Conservation of Resources Theory

#### 3.6.1. Resistance Resources (RRs)

Resistance Resources (RRs) are factors that aid individuals in managing stress and maintaining health [2,3]. In other words, access to stronger resources is associated with lower levels of psychological distress, due to its influence on high perceptions of coherence. Conversely, fewer resources are associated with increased psychological distress, leading to poor well-being [47]. RRs are categorized into two main types according to Antonovsky: Specific Resistance Resources and Generalized Resistance Resources.

Specific Resistance Resources (SRRs) are context-dependent resources tailored to address particular stressors or challenges. If one has a math test, for instance, SRRs might include study guides, tutoring sessions, and practice problems. Generalized Resistance Resources (GRRs) are broader resources that are not tied to a particular challenge but contribute to overall resilience and well-being, for instance, a supportive family, a positive attitude, or living in a homogenous community.

#### 3.6.2. Conservation of Resources (COR) Theory and Sense of Coherence (SOC)

Hobfoll’s Conservation of Resources (COR) theory posits that changes, especially after traumatic events, feel threatening because of the loss or possible loss of valuable resources [21]. This idea fits well with Antonovsky’s salutogenic model, which emphasizes the importance of Generalized Resistant Resources (GRRs) in preparing individuals for a wide array of stressors in both internal and external environments [3]. While a pathogenic orientation—the dichotomous position that humans are either well or sick—focuses on Specific Resistant Resources (SRRs) for specific purposes, such as specific illnesses, on the other hand, the salutogenic orientation and, consequently, the SOC concept rely more heavily on broader supports—GRRs. However, GRRs can facilitate access to seemingly endless SRRs for different life scenarios [2]. For instance, having a supportive family (GRR) can help one get through many different challenges, whether it is studying for a test, dealing with an illness, or recovering from a traumatic event.

**Figure 2:**
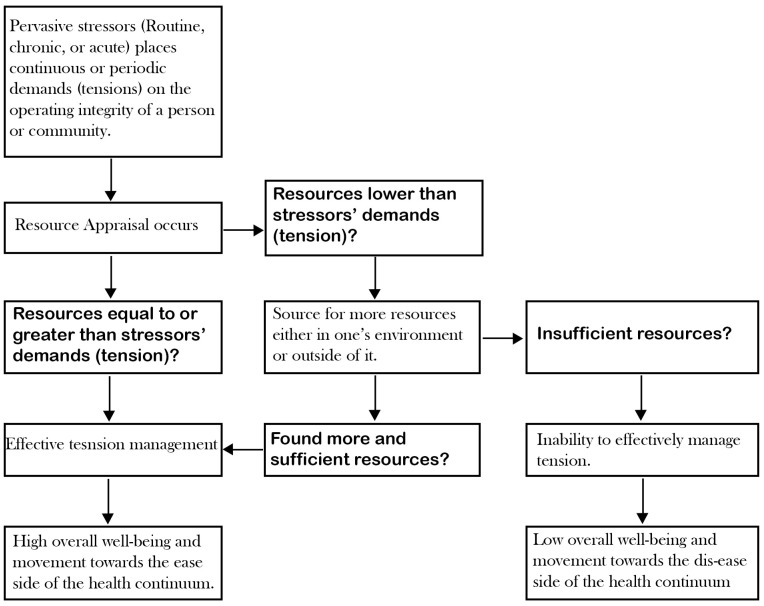
How resource appraisal mediates tension management (adapted from a combined framework of salutogenesis and COR [2,3,21].

Hobfoll’s COR model also suggests that individuals strive to obtain, retain, foster, and protect resources [21], with stress occurring when there is a lack of resources to engage existing tensions or an anticipation of potential resource deficiency in response to future tensions. Essentially, it means that there is a continuous appraisal of available resources relative to the tension imposed by various stressors. As shown in Figure 2, tension management is made possible when the resources available are greater than the tension demands.

**Figure 3:**
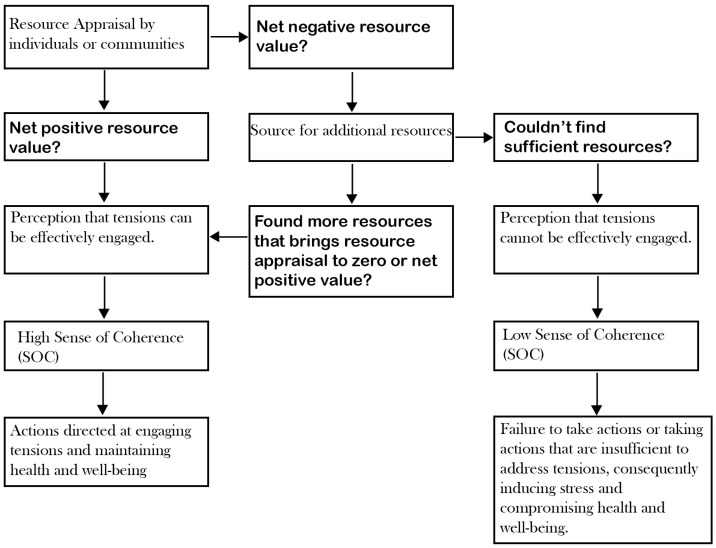
How resource appraisal relates to the SOC.

From Figure 2, the perception of resource availability significantly influences an individual’s sense of coherence through the net value of resource appraisal that occurs consciously or subconsciously [21]. This is further illustrated in Figure 3, which demonstrates the importance of resource sufficiency. A zero or net positive resource value—where available resources meet or exceed the demands of stressors—strengthens the SOC. In simpler terms, the more resources available to manage tensions, the stronger the SOC. Conversely, a net negative resource value, where resources fall short of what is needed to address stressors, weakens the SOC. This means that, when resources are significantly limited, the SOC diminishes, leaving individuals more susceptible to the adverse effects of stress.

As shown in Figure 3, the perception of available Resistant Resources (RRs) relative to tensions strengthens people’s confidence and propels people to act to maintain or improve their position on the health continuum. Hence, the relationship between RRs and tension management, mediated by the SOC, is vital for understanding how available resources contribute to overall well-being and resilience.

#### 3.6.3. Impact of Resistant Resources (RRs) on SOC

Hobfoll distinguishes between “raw resources” and “evaluated resources” to highlight the importance of individual interpretation in transforming available resources into assets that promote health [21]. This distinction aligns with Antonovsky’s concept of GRRs, where resources such as social capital exist in an unprocessed state. Through cognitive appraisal, these “raw resources” are transformed into resources with tangible impacts on health outcomes [3,21].

Raw resources are resources that, in their initial form, may not provide clear support. For example, a group of friendly neighbors may be seen as simply people who live nearby without much interaction. On the other hand, “evaluated resources” are raw resources that have been processed through cognitive interpretation and recognized as valuable. This could be the realization that those friendly neighbors might offer support during difficult times. This cognitive shift helps build GRRs, similar to how recognizing the support of neighbors can enhance one’s social capital.

Hobfoll also emphasizes that personal values play a significant role in interpreting raw resources [21]. These values shape how individuals assess the usefulness of available resources. Understanding the role of values highlights the need for interventions that are tailored to the value systems of the target population. For instance, if people in a community value strong family ties, they are more likely to recognize the importance of family support as a resource. The more the available raw resources align with the community’s values, the more they can be evaluated and transformed into beneficial resources, which ultimately strengthens their SOC.

The process by which RRs strengthen the SOC can be conceptualized as a continuous mechanism. Within this mechanism, individuals constantly transform raw resources into evaluated resources to address stressors. According to Antonovsky, this transmuting process is characterized by a series of life experiences marked by three elements: consistency, load balance, and participation in outcome [2,3]. When these three elements are present, they help strengthen the SOC.

Consistency manifests as a structured, predictable, and explicable channel for meeting stressor-imposed demands. Consistency enhances the comprehensibility component of SOC by fostering an environment perceived as predictable and understandable, thereby mitigating anxiety and amplifying cognitive clarity [2]. Load balance involves a continuous effort to maintain equilibrium between underload and overload, resulting in a state of heterostatic equilibrium. This strengthens the manageability component of SOC, as the goal is a state where individuals are neither overwhelmed nor underwhelmed [2]. The participation in outcome concept ties back to the importance of values. When individuals perceive congruence between their environment and their personal, social, and cultural values, and they can be involved in the decisions related to what resources are added to their reservoir, their motivation to engage with stressors’ demands is heightened [2,3,48].

Understanding and leveraging these three cognitive mechanisms can inform the planning and design of interventions aimed at strengthening the SOC. Such interventions could provide platforms for life experiences that are consistent, help to maintain load balance, and involve the extensive participation of users in the process. In essence, the concepts of consistency, load balance, and participation in outcomes strengthen the core components of SOC—comprehensibility, manageability, and meaningfulness—by helping individuals understand, manage, and find purpose in their resources and stressors. Together, they enhance the process of identifying raw resources and transforming them into evaluated resources, which fosters resilience and overall well-being.

## 4. Discussion

While the previous section explored how individual and community resources contribute to building resilience, it is equally important to consider the broader policy and systemic frameworks that enable and support establishing a sense of coherence. As such, addressing resilience and health in disaster-prone communities requires a closer examination of disaster recovery research and its intersection with policy development. Effective policymaking is vital for facilitating coordinated responses and ensuring the success of interventions across the various levels of ecological systems to increase opportunities for communities to establish a strong sense of coherence.

### 4.1. Disasters as Focusing Events

Central to this discussion around policy and frameworks in the disaster context are John Kingdon’s idea of focusing events and Thomas Birkland’s concept of policy change after disasters [17,49]. These two concepts, in particular, help clarify the mechanisms and strategies that promote adaptive resilience and well-being in post-disaster environments.

Kingdon’s idea of focusing events suggests that certain occurrences, such as disasters, can rapidly push issues to the forefront of policy agendas [16,17,49]. Disasters often serve as powerful focusing events because they expose existing policy inadequacies or gaps, create a sense of urgency for immediate action, attract significant media and public attention, and create opportunities for policy entrepreneurs to advocate for change [16,17]. Birkland’s work extends this concept, specifically examining how disasters can drive policy change [16,17]. He emphasizes the potential for instrumental, social, and political policy learning in the aftermath of disasters. Social policy learning is critical to the discussion around developing a sense of coherence for communities because it shapes the social construction of policies or programs [17]. Such learning can lead to policy changes that reshape societal views on risk, resilience, and recovery.

Regardless of the impacted community, the post-disaster recovery phase presents a unique “policy window”—an opportunity to implement measures that address the community’s immediate needs and long-term adaptive capacity. This window allows for an easier and more timely introduction of sustainable recovery efforts that can mitigate both the immediate and long-term impacts of disasters while building future resilience, compared to times when the issues do not seem as urgent or necessary.

It is important to recognize that the effects of disasters extend far beyond the initial impact. Research suggests that loss events associated with disasters are not discrete points in time but prolonged processes [13,21]. The phenomenon of stress proliferation, when initial stressors lead to additional stressors over time, in the wake of disasters underscores the importance of post-disaster planning and design that promotes long-term well-being. During this period following the disaster event, affected individuals often experience a pervasive sense of disorientation [13] and a widespread and deep-seated feeling of being unsettled or ungrounded caused by a disruption of routines, familiarity, safety, and control. Thus, disaster survivors often actively seek both internal and external resources to cope with the myriads of stressors that emerge over time, many of which are exacerbated by the ripple effects of the initial disaster. This process of resource-seeking and coping—or seeking a sense of coherence as previously defined—occurs against a backdrop of prolonged stress and uncertainty, further emphasizing the importance of effective policy interventions to help ward off stressors from the beginning or better still bolster the capacity to engage them when they impose tensions.

Viewing disasters through the lenses of both focusing events and policy windows can provide valuable insights for policymakers, community leaders, and researchers. These perspectives emphasize the potential for meaningful policy change in the wake of disasters and highlight the importance of leveraging post-disaster opportunities to promote a sense of coherence and consequently enhance community resilience and well-being. Future research and policy initiatives could thus focus on developing strategies to effectively utilize post-disaster recovery rebuilding to address the complex and prolonged nature of stress and recovery in disaster-prone environments.

### 4.2. Opportunities for Policy Change and Learning

The post-disaster transitional stage plays a pivotal role in the dynamic and iterative cycle of disaster management [14]. Gil-Rivas and Kilmer conceptualize the post-disaster recovery period as both an aftermath and a potential prelude to future occurrences, creating a critical juncture where the efficacy of adaptation strategies, utilization of resilient resources, and commitment to ongoing adaptation become particularly salient [18]. As such, this period post-disaster is a key moment for considering the SOC.

Major disasters often create opportunities for significant policy reforms that enhance both SRRs and GRRs. These disaster events frequently expose the existing resource deficiencies, prompting reassessment and reallocation. This exposure of weaknesses may catalyze policy changes to address identified deficiencies while fostering identified strengths. As Birkland observes, many individuals do not seriously consider disasters until one occurs [17]. This wait-and-see approach underlines the potential for natural disasters to serve as catalysts for targeted interventions, facilitated by policies whose necessity becomes undeniable due to the heightened attention these events generate.

Of particular interest in this post-disaster context is the idea of resource evaluation and value appraisal [21]. The post-disaster period provides a unique opportunity for survivors to reassess their priorities and recognize the importance of previously undervalued aspects of their lives. For instance, the unfolding events after a natural disaster may make survivors begin to see the importance of their living environments, such as the fondness they have developed for their neighbors, whom they had thought were annoying, or the peculiar layout of their communities that they had not paid attention to before. Environmental resources perceived as lost or unavailable, depending on how they shift the perceptive value of resource reservoir, could be the difference between well-recovered and poorly recovered disaster survivors. Therefore, interactions with and data gathering in affected communities can yield valuable insights into the factors that are perceived as most critical during recovery. This population-based data would help to curate a survivor-informed catalog of raw resources that could be introduced into rebuilding, which could then be transformed into evaluated resources by survivors for resisting different stressors’ demands in recovery and in the next acute disaster.

Although this paper primarily focuses on insights from urban and developed contexts, it is essential to acknowledge the unique challenges posed by socioeconomic vulnerabilities in developing economies. In these settings, disaster response often encounters compounded obstacles arising from resource scarcity, fragile infrastructure, and fragmented policy frameworks, which collectively hinder effective recovery efforts [50,51]. The built environment, already fragile in many of these regions, exacerbates susceptibility to disaster impacts, frequently resulting in prolonged and uneven recovery trajectories [52]. Integrating adaptive resilience strategies that emphasize community engagement and individual agency is vital for addressing these persistent challenges.

In this regard, the sense of coherence (SOC) framework still holds significant potential. By systematically targeting critical resource deficits through its core components—comprehensibility, manageability, and meaningfulness—the SOC approach offers a structured alternative to ad hoc or generalized interventions. Recovery asset mapping, guided by these three components, ensures more focused and sustainable recovery efforts, particularly in resource-constrained environments where financial limitations demand the judicious allocation of structural, systemic, and fiscal resources. In essence, interventions guided by the SOC framework can focus on critical areas of need, ensuring that scarce resources are used effectively to maximize impact.

### 4.3. Opportunities for Planning and Design: Anchors of Coherence

As purported by Gil-Rivas and Kilmer, the post-disaster stage may mark the aftermath of a recent disaster, but it is simultaneously a pre-event stage for future disasters [18]. In this sense, actions taken during this period help survivors recover from past events and prepare them for future harm. The recovery stage is also a time to institute sustainable and improved pre-event recovery plans for future disasters [53]. Here, emphasizing the importance of sense of coherence (SOC), stressors, and policy, the role of planning and design in shaping resilient communities becomes even more vital, especially in the post-disaster environment.

Planning and design act as practical extensions of policy goals, creating tangible interventions that address psychosocial and environmental needs in disaster-prone communities. The environment, hence, plays a pivotal role as a determinant of health [54], serving as an active agent in shaping human experiences and well-being. In essence, the built environment and its design can serve as vital “anchors of coherence”, helping individuals and communities traverse disaster recovery effectively by promoting stability and, consequently, well-being. Anchors of coherence are features within the environment that enable individuals and communities to make sense of, connect with, and derive meaning from their surroundings [13]. These anchors, which may include tangible structures or intangible cultural and emotional elements, serve as platforms for strengthening the sense of coherence (SOC).

While immediate emergency shelter remains a top priority in the aftermath of a disaster, the true value of design becomes increasingly apparent during the transitional stages of rebuilding and resettlement [14]. The challenge for planners and designers is how engagement with the built environment can foster experiences that make life comprehensible, manageable, and meaningful, in support of an increased SOC to support community resilience. Strategically planned anchors in the built environment can function as psychological, emotional, and autobiographical markers that provide a sense of rootedness and coherence. For example, preserving a local landmark can create familiarity and a sense of control amid uncertainty. Similarly, designing communal spaces that encourage social interaction or incorporating culturally significant symbols into recovery efforts can serve as powerful touchpoints for comfort, security, and identity reconstruction after disruptive events. These elements not only address immediate recovery needs but also lay a foundation for long-term well-being, helping communities prepare for future challenges.

Plowright and Adhya offer a valuable framework for understanding how people cognitively engage with their built environment [55]. They outline two ways to understand coherence through design. First is formal coherence. This strategy evaluates whether the environment is laid out in a way that meets the needs of the people, thereby creating a sense of comprehensible and manageable lives. The second is event coherence. Event coherence ensures that the layout of the environment reflects the underlying ways of life and values of the community, inherently referring to meaningfulness. Through these ways of understanding coherence, design must facilitate life experiences that allow Resistant Resources to manifest through both formal and event coherence, to strengthen the overall sense of coherence (SOC).

Furthermore, it is during the post-disaster rebuilding phase, or even the intentional planning for prevention process, that planners and designers can imbue a sense of resource net gain in the post-disaster context. The process of resource evaluation in the aftermath of a disaster is continuous and dynamic. As such, the goal of planners and designers, either after or before a disaster, should be to create environments that incrementally help people increase their perception of resource replenishment. This type of support can be made possible by leveraging the psychological, emotional, and autobiographical anchors of coherence, as identified through interactions with the affected communities.

This approach to supportive planning and design recognizes the long-term nature of disaster recovery and the importance of sustained support for affected communities. It becomes increasingly clear that the built environment is not just a passive backdrop to human experiences, but an active participant in shaping our responses to stress and adversity, particularly through the lens of disaster support. By harnessing the power of thoughtful design and planning, environments can be created that support individuals and communities in their journey toward recovery and resilience in the face of disasters through SOC-promoting anchors that have been intentionally incorporated into disaster recovery planning.

### 4.4. Future Research: Methodological Considerations

The study of salutogenesis, and more specifically, the sense of coherence (SOC) and its application in disaster recovery contexts, presents unique methodological challenges and opportunities for future research. Vaandrager and Kennedy highlight a critical methodological gap in the current research landscape—the predominance of quantitative studies in salutogenesis research [12]. While these quantitative studies have provided valuable insights, they often fall short of capturing the rich, contextual nuances of human experiences in disaster-prone environments. The call for more qualitative inquiry opens up exciting avenues for researchers to explore the depth and breadth of community resilience in ways that numbers alone cannot capture. Longitudinal and cross-sectional case studies could provide valuable data that better illuminate the human experience of disaster.

Erikson and Peek emphasize the power of first-hand accounts in understanding the human experience of disasters [13]. This data collection approach aligns with qualitative methodologies of ethnography and narrative inquiry [56,57]. Gathering survivors’ accounts of recovery can enable researchers to gain invaluable insights into the lived experiences of disaster survivors and, in doing so, uncover the subtle ways in which the SOC manifests and evolves in real-world settings. Participatory action research (PAR) presents another promising avenue for disaster recovery studies. This engagement-based approach generates knowledge and empowers communities to actively engage in their own recovery process [58]. In addition, mixed-method approaches that combine the quantitative measures of SOC with qualitative explorations of community experiences can provide a more comprehensive understanding of resilience processes. Such approaches allow researchers to triangulate findings, thus enhancing the validity and reliability of their conclusions.

As we embrace these diverse methodologies, however, it is vital to remain mindful of potential biases and limitations. Cultural sensitivity is imperative in the SOC research, particularly in communities affected by disasters [59]. While seeking in-depth, contextualized data, researchers must be aware of local customs, beliefs, and values that may influence how the SOC is conceptualized and expressed in different cultural contexts. Moreover, disaster recovery studies face unique challenges, such as the potential for researcher bias due to the emotional nature of the subject matter, difficulties in establishing pre-disaster baselines, and the ethical considerations of conducting research with vulnerable populations. Acknowledging these limitations is key to maintaining the integrity of the research and interpreting findings responsibly.

## 5. Conclusions

The evolving disaster recovery and resilience research terrain needs an integrated understanding of how individuals and communities adapt to and recover from acute events. This paper has highlighted the opportunity for the sense of coherence (SOC) concept to be a pivotal construct for enhancing resilience and long-term adaptability in the face of natural disasters. By drawing on the seminal works of Antonovsky, Hobfoll, Birkland, and other key scholars, this paper has explored the nature of stressors, the mechanisms of SOC, and the critical role of Resistance Resources (RRs).

Several important insights emerge from this exploration. First, the intuitive, “sense-making” approach to managing chaotic situations remains a key challenge. As Davoudi et al. emphasized, understanding this human dimension is essential for developing effective resilience strategies [6]. Future research should therefore integrate qualitative data, such as survivor narratives, alongside quantitative analyses to comprehensively understand how resilience factors assist individuals and communities in disaster recovery [11,12].

Much of disaster research has focused on understanding natural hazards, but equally important are the survivors who directly influence recovery outcomes. Whether through community-driven rebuilding efforts, local problem-solving, or psychological resilience, survivors’ actions, decisions, and adaptive capacities directly influence how recovery unfolds. This underscores the need to study disaster survivors just as thoroughly as the natural hazards themselves because they are equally—if not more—important in determining the success of recovery efforts.

In disaster recovery, combining technological fixes (engineering solutions) and cognitive fixes (disseminating disaster-related information) has not been sufficient. A more impactful approach involves structural fixes, creating intentional interventions that shape life experiences, which, in turn, influence the long-term recovery behavior. Structural fixes, driven by urban design and architecture, can foster environments where people internalize positive recovery behaviors. While changes in the SOC or attitude are generally slow, it is responsive to consistent life experiences, whether positive or negative, rather than one-time interventions [2,44].

Extensive information was provided on the SOC as a critical resilience factor that can enhance the human dimensions of resilience in disaster-prone communities for future research. This paper identifies gaps in understanding the community-level resources that bolster the SOC. Research should focus on mapping design assets that enhance the collective sense of coherence [8,60,61,62]. As noted by Heberlein, collective norms function much like magnets, pulling behavior toward what is considered “average” or “normal” [44]. Interventions grounded in collective norms have the potential to uplift the entire community regardless of where individuals fall on the SOC continuum by providing support to all members, and with consistency over time, those at the lower end of the SOC spectrum may gradually be “pulled up”, contributing to more inclusive and sustained recovery.

Additionally, the post-disaster recovery phase presents a unique “policy window” for policy reform and learning—an opportunity to implement measures that address the community’s immediate needs and long-term adaptive capacity [16,17,50]. Policy-driven structural fixes that encourage recovery behaviors can compel individuals to participate in recovery efforts. As long as these efforts yield visible improvements, individuals tend to align their behavior with collective recovery norms, regardless of their SOC. In this way, both structural and social drivers act as “magnets”, pulling behavior toward recovery and creating a cycle of action and reinforcement that drives long-term resilience.

Finally, researchers must expand their approach to salutogenesis. There is a need to explore how built environments—beyond biophilic designs—can improve health outcomes even in the absence of natural views [63]. This broader application of salutogenic principles can significantly contribute to resilient and adaptive communities.

Bridging the identified research gaps between community health, resilience, and design and planning, while also advancing the understanding of the value of incorporating SOC into resilience, requires a multidisciplinary approach. Explorations into this complex issue must integrate insights from health, public policy, psychology, urban planning, architecture, and environmental design. Through the SOC or similar frameworks, communities can be positioned to better traverse the challenges posed by natural disasters and achieve more resilient and equitable outcomes. Future research should continue to explore this complex interplay of resources, individual perceptions, and environmental factors to develop strategies that support resilience and well-being in the face of persistent challenges.

## Figures and Tables

**Table 1:** Differences between salutogenesis and pathogenesis.

	Salutogenesis	Pathogenesis
View of stressors and chaos	Acknowledges stressors as an inherent aspect of life and emphasizes the importance of continuous adaptability in the face of stressors	Views stressors as anomalies in nature and perceives the inherent state of human beings as stable
View of health	Views health as a continuum between extremes of disease and ease	Views health as a dichotomy of sick or healthy
What is worth studying	Aims to identify and understand why certain individuals or communities maintain good health despite continuous exposure to factors that typically induce stress	Seeks to comprehend why individuals or communities become unwell and how to stop the factors that cause vulnerabilities or diseases
Key to health	The key to health lies in a sense of coherence (SOC) that is fostered by the resources in one’s personal or community reservoir.	Follows the “magic bullet” approach, addressing one disease with one cure

Source: [2,3].

## Data Availability

No new data was created or analyzed in this study. Data sharing is not applicable to this article.

## References

[1] Maslow A.H. (1943). A theory of human motivation. Psychol. Rev..

[2] Antonovsky A. (1979). Health, Stress, and Coping. Jossey-Bass Publishers. https://www.proquest.com/docview/60038331?pq-origsite=summon&sourcetype=Books.

[3] Antonovsky A. (1987). Unraveling the Mystery of Health: How People Manage Stress and Stay Well, 1st ed. Jossey-Bass. https://books.google.com/books/about/Unraveling_the_Mystery_of_Health.html?id=6HVHAAAAMAAJ.

[4] Mittelmark M.B., Mittelmark M.B., Bauer G.F., Vaandrager L., Pelikan J.M., Sagy S., Eriksson M., Lindström B., Meier Magistretti C. (2022). Stressor Appraisal on a Pathway to Health: The Role of the Sense of Coherence. The Handbook of Salutogenesis.

[5] Vinje H.F., Langeland E., Bull T., Mittelmark M.B., Sagy S., Eriksson M., Bauer G.F., Pelikan J.M., Lindström B., Espnes G.A. (2017). Aaron Antonovsky’s Development of Salutogenesis, 1979 to 1994. The Handbook of Salutogenesis.

[6] Davoudi S., Shaw K., Haider L.J., Quinlan A.E., Peterson G.D., Wilkinson C., Fünfgeld H., McEvoy D., Porter L., Davoudi S. (2012). Resilience: A Bridging Concept or a Dead End? ‘Reframing’ Resilience: Challenges for Planning Theory and Practice Interacting Traps: Resilience Assessment of a Pasture Management System in Northern Afghanistan Urban Resilience: What Does it Mean in Planning Practice? Resilience as a Useful Concept for Climate Change Adaptation? The Politics of Resilience for Planning: A Cautionary Note: Edited by Simin Davoudi and Libby Porter. Plan. Theory Pract..

[7] Eriksson M., Mittelmark M.B., Bauer G.F., Vaandrager L., Pelikan J.M., Sagy S., Eriksson M., Lindström B., Meier Magistretti C. (2022). The Sense of Coherence: The Concept and Its Relationship to Health. The Handbook of Salutogenesis.

[8] Généreux M., Roy M., O’Sullivan T., Maltais D., Mittelmark M.B., Bauer G.F., Vaandrager L., Pelikan J.M., Sagy S., Eriksson M., Lindström B., Meier Magistretti C. (2022). Salutogenesis as a Framework for Social Recovery After Disaster. The Handbook of Salutogenesis.

[9] Montesanti S., Walker I., Chan A.W.H. (2022). Editorial: Improving disaster health outcomes and resilience through rapid research: Implications for public health policy and practice. Front. Public Health.

[10] Eriksson M. (2007). Unravelling the Mystery of Salutogenesis: The Evidence Base of the Salutogenic Research as Measured by Antonovsky’s Sense of Coherence Scale.

[11] Aldrich D. (2011). The Power of People: Social Capital’s Role in Recovery from th1995 Kobe Earthquake. Nat. Hazards.

[12] Vaandrager L., Kennedy L., Mittelmark M.B., Bauer G.F., Vaandrager L., Pelikan J.M., Sagy S., Eriksson M., Lindström B., Meier Mag-istretti C. (2022). The Application of Salutogenesis in Communities and Neighborhoods. The Handbook of Salutogenesis.

[13] Erikson K., Peek L.A. (2022). The Continuing Storm: Learning from Katrina.

[14] Charlesworth E., Fien J. (2022). Design and Disaster Resilience: Toward a Role for Design in Disaster Mitigation and Recovery. Architecture.

[15] Nugent N.R., Sumner J.A., Amstadter A.B. (2014). Resilience after trauma: From surviving to thriving. Eur. J. Psychotraumatol..

[16] Birkland T.A. (1998). Focusing Events, Mobilization, and Agenda Setting. J. Pub. Pol..

[17] Birkland T.A. (2006). Lessons of Disaster: Policy Change After Catastrophic Events.

[18] Gil-Rivas V., Kilmer R.P. (2016). Building Community Capacity and Fostering Disaster Resilience. J. Clin. Psychol..

[19] Manyena B. (2013). Disaster event: Window of opportunity to implement global disaster policies?. Jàmbá J. Disaster Risk Stud..

[20] Solecki W.D., Michaels S. (1994). Looking through the postdisaster policy window. Environ. Manag..

[21] Hobfoll S.E. (1988). The ecology of stress. The Ecology of Stress.

[22] Selye H. (1956). The Stress of Life.

[23] Gunderson L., Holling C.S. (2002). Panarchy: Understanding Transformations in Human and Natural Systems.

[24] Wheaton B. (1999). The nature of stressors. A Handbook for the Study of Mental Health: Social Contexts, Theories, and Systems.

[25] Pearlin L.I., Schieman S., Fazio E.M., Meersman S.C. (2005). Stress, Health, and the Life Course: Some Conceptual Perspectives. J. Health Soc. Behav..

[26] Aneshensel C.S., Rutter C.M., Lachenbruch P.A. (1991). Social structure, stress, and mental health: Competing conceptual and analytic models. Am. Sociol. Rev..

[27] Phillips B.D., Mincin J. (2023). Disaster Recovery.

[28] Kasperson R.E., Renn O., Slovic P., Brown H.S., Emel J., Goble R., Kasperson J.X., Ratick S. (1988). The Social Amplification of Risk: A Conceptual Framework. Risk Anal..

[29] Bahmani H., Zhang W. (2022). Why Do Communities Recover Differently after Socio-Natural Disasters? Pathways to Com-prehensive Success of Recovery Projects Based on Bam’s (Iran) Neighborhoods’ Perspective. Int. J. Environ. Res. Public Health.

[30] World Health Organization (WHO) (1948). “constitution-en.pdf,” Presented at the International Health Conference, New York. https://www.who.int/about/governance/constitution.

[31] Stricker J., Jakob L., Pietrowsky R. (2023). Associations of continuum beliefs with personality disorder stigma: Correlational and experimental evidence. Soc. Psychiatry Psychiatr. Epidemiol..

[32] Makowski A.C., Schomerus G., von dem Knesebeck O. (2021). Public Continuum Beliefs for Different Levels of Depression Severity. Front. Psychiatry.

[33] Escandón K., Rasmussen A.L., Bogoch I.I., Murray E.J., Escandón K., Popescu S.V., Kindrachuk J. (2021). COVID-19 false dichotomies and a comprehensive review of the evidence regarding public health, COVID-19 symptomatology, SARS-CoV-2 transmission, mask wearing, and reinfection. BMC Infect. Dis..

[34] Holling C.S. (1973). Resilience and Stability of Ecological Systems. Annu. Rev. Ecol. Syst..

[35] Folke C., Carpenter S.R., Walker B., Scheffer M., Chapin T., Rockström J. (2010). Resilience thinking: Integrating resilience, adaptability and transformability. Ecol. Soc..

[36] Waller M.A. (2001). Resilience in ecosystemic context: Evolution of the concept. Am. J. Orthopsychiatry.

[37] Stenlund S., Mâsse L.C., Stenlund D., Sillanmäki L., Appelt K.C., Koivumaa-Honkanen H., Rautava P., Suominen S., Patrick D.M. (2023). Do Patients’ Psychosocial Characteristics Impact Antibiotic Prescription Rates?. Antibiotics.

[38] Weitzel E.C., Glaesmer H., Hinz A., Zeynalova S., Henger S., Engel C., Löffler M., Reyes N., Wirkner K., Witte A.V. (2022). What Builds Resilience? Sociodemographic and Social Correlates in the Population-Based LIFE-Adult-Study. Int. J. Environ. Res. Public Health.

[39] Levine S. (2003). Psychological and social aspects of resilience: A synthesis of risks and resources. Dialogues Clin. Neurosci..

[40] Kaplan S. (1983). A Model of Person-Environment Compatibility. Environ. Behav..

[41] Burgess A., Wardman J., Mythen G. (2018). Considering risk: Placing the work of Ulrich Beck in context. J. Risk Res..

[42] Slovic P., Finucane M.L., Peters E., MacGregor D.G. (2004). Risk as analysis and risk as feelings: Some thoughts about affect, reason, risk, and rationality. Risk Anal..

[43] Beck U. (1992). Risk Society; Towards a New Modernity. https://books.google.com/books/about/Risk_Society.html?id=QUDMaGlCuEQC.

[44] Heberlein T.A. (2012). Navigating Environmental Attitudes.

[45] Langeland E., Wahl A.K., Kristoffersen K., Nortvedt M.W., Hanestad B.R. (2007). Sense of coherence predicts change in life satisfaction among home-living residents in the community with mental health problems: A 1-year follow-up study. Qual. Life Res..

[46] Hikichi H., Shiba K., Aida J., Kondo K., Kawachi I. (2023). Association between sense of coherence and health and well-being among older survivors of a natural disaster: A prospective outcome-wide study. Sci. Rep..

[47] Pepe A., Cavazzoni F., Addimando L., Jaradah A., Obaid H., Veronese G. (2021). Wellbeing, symptoms of trauma, and personal resources in Palestinian professional helpers: A cross-sectional quantitative survey. Lancet.

[48] Antonovsky A. (1993). Complexity, conflict, chaos, coherence, coercion and civility. Soc. Sci. Med..

[49] Kingdon J.W. (1995). Agendas, Alternatives, and Public Policies.

[50] Global Facility for Disaster Reduction and Recovery (GFDRR) Disaster Recovery Framework Guide; World Bank: 2020. https://www.gfdrr.org/en/publication/disaster-recovery-framework-guide-0.

[51] The Commonwealth A New Programme of Action for Commonwealth LDCs. Commonwealth..

[52] United Nations Climate Change Secretariat (2018). Considerations Regarding Vulnerable Groups, Communities and Ecosystems in the Context of the National Adaptation Plans.

[53] Smith G. (2011). Planning for Post-Disaster Recovery: A Review of the United States Disaster Assistance Frame-Work.

[54] Azzopardi-Muscat N., Brambilla A., Caracci F., Capolongo S. (2020). Synergies in Design and Health. The role of architects and urban health planners in tackling key contemporary public health challenges. Acta Bio Medica Atenei Parm..

[55] Plowright P.D., Adhya A. (2022). Urban Design Made by Humans: A Handbook of Design Ideas.

[56] Caine V., Clandinin D.J., Lessard S. (2022). Narrative Inquiry: Philosophical Roots.

[57] Emerson R.M., Fretz R.I., Shaw L.L. (2011). Writing Ethnographic Fieldnotes, Second Edition. Chicago Guides to Writing, Editing, and Publishing.

[58] Chevalier J.M. (2019). Participatory Action Research: Theory and Methods for Engaged Inquiry.

[59] Daoud N., Braun-Lewensohn O., Eriksson M., Sagy S. (2014). Sense of coherence and depressive symptoms among low-income Bedouin women in the Negev Israel. J. Ment. Health.

[60] Leviton L.C., Snell E., McGinnis M. (2000). “Urban Issues in Health Promotion Strategies. Am. J. Public Health.

[61] Maass R., Lindström B., Lillefjell M. (2017). Neighborhood-resources for the development of a strong SOC and the im-portance of understanding why and how resources work: A grounded theory approach. BMC Public Health.

[62] van Sint Fiet A., de la Rie S., van der Aa N., Bloemen E., Wind T. (2022). The relevance of social capital and sense of coherence for mental health of refugees. SSM Popul. Health.

[63] Golembiewski J.A., Mittelmark M.B., Bauer G.F., Vaandrager L., Pelikan J.M., Sagy S., Eriksson M., Lindström B., Meier Magistretti C. (2022). Salutogenic Architecture. The Handbook of Salutogenesis.

